# *Rhizobium radiobacter* pseudo-outbreak linked to tissue-processing contamination

**DOI:** 10.1017/ash.2023.456

**Published:** 2023-10-20

**Authors:** Rebecca A. Stern, Kelly C. Byrge, Thomas R. Talbot

**Affiliations:** Division of Infectious Diseases, Department of Medicine, Vanderbilt University Medical Center, Nashville, TN, USA

## Abstract

A cluster of *Rhizobium radiobacter* isolates isolated from six unique surgical tissue cultures prompted an investigation ultimately identifying a pseudo-outbreak linked to errant laboratory tissue processing with contaminated, nonsterile saline. Timely response and multidisciplinary collaboration led to tangible system-level interventions and avoidance of unnecessary antibiotic exposures.

## Introduction

*Rhizobium radiobacter*, formerly Agrobacterium, is an environmental aerobic Gram-negative bacillus infrequently associated with human disease. Rare pseudo-outbreaks have been reported, typically bacteremia related to contaminated saline.^1,2^ Risk factors for opportunistic infection with *R. radiobacter* include the presence of an intravenous catheter or device, neutropenia, and hematologic or solid organ malignancy.^3,4^ Herein we describe investigation of a pseudo-outbreak of *R. radiobacter* involving surgical patients due to tissue culture contamination by an error in laboratory processing technique.

## Methods

In January 2023, six patients with *R. radiobacter* isolated in surgical tissue specimens were identified in an acute care hospital within a 16-d span (Table 1). Microbiologic diagnosis was made using MALDI-TOF mass spectrometry. Upon learning of the second case of Rhizobium from clinical infectious disease consulting physicians, there was rapid recruitment of a multidisciplinary team to conduct an epidemiologic investigation for a potential outbreak, including the Infection Prevention and Control Committee (IPC), hospital and laboratory administration, microbiology, surgery, central sterile processing, and facilities operations.

**Table 1.** Demographic and clinical characteristics of patients with tissue culture positive for Rhizobium

Patient No.	Age (yrs)	Sex	Comorbidities	Procedure	Specimen/Tissue Source	Inpatient Ward Location	Culture Date	Other Organisms Isolated	Treatment targeted to Rhizobium
1	75	M	Diabetes, neuropathy, PAD	Trans-metatarsal amputation with flap	Right foot 1st metatarsal bone	Medical floor 5	1/9/23	No	Yes; Ciprofloxacin, Flagyl
2	69	M	ESRD on hemodialysis	Left upper extremity AVG partial explant, patch angioplasty	Left upper extremity AVF graft; wound/tissue spec	Medical floor 6	1/11/23	Staphylococcus epidermidis in perma-cath blood cultures (1/11/23)	Yes; Cefepime, Vancomycin
3	54	F	None	VATS	Right lung empyema	Medical floor 6	1/20/23	Citrobacter freundii in pleural fluid (1/16/23)	Yes; Cefepime
4	42	M	None	VATS	Left lung empyema	Medical floor 6	1/20/23	No	Yes; Piperacillin-tazobactam
5	68	F	DVT with IVC filter	Revision THA	Right hip	Medical floor 4	1/24/23	No	No
6	60	M	Diabetes	Revision TKA	Left knee	Medical floor 4	1/24/23	Staphylococcus lugdunensis in knee tissue (1/24/23)	No

Note. PAD, peripheral arterial disease; ESRD, end-stage renal disease; AVF, arteriovenous fistula; DVT, deep venous thrombosis; IVC, inferior vena cava; VATS, video-assisted thoracic surgery; THA, total hip arthroplasty; TKA, total knee arthroplasty; CoNS, coagulase-negative Staphylococcus.

### Patient characteristics

A chart review of clinical and microbiologic data was performed for all six cases. Five patients underwent different types of surgery by separate surgeons (Table 1), and only one patient had a central line. No other unifying symptoms, demographics, locations, or exposures were initially apparent. Rhizobium growth was detected only in tissue cultures of all cases and was not isolated from cultures of other body fluids, bone, or swab samples.

### Epidemiologic investigation

A review of all Gram-negative bacilli isolated in the microbiology lab (culture-based or MALDI-TOF) over the six months prior to identification of Rhizobium (July 2022 through January 2023) showed no other Rhizobium isolates, thereby establishing a clear timeline of onset. Inquiry was made to the state health department, which reported no other similar local or regional outbreaks. Source investigation began by mapping out supplies and processes used in the operating rooms (OR), as well as specimen transportation and processing procedures in the microbiology laboratory. Environmental samples were obtained of sterile saline from the OR, saline from OR warmers, air from the three ORs where patients had undergone surgery, and tissue specimen cups. Samples in the microbiology laboratory were obtained from the tissue grinder and cotton-tipped applicator used to transfer samples from specimen cups to tissue vials. Laboratory personnel were interviewed regarding procedures for tissue culture processing.

## Results

### Procedures and environmental investigation

None of the environmental sample cultures from OR supplies grew Rhizobium. No issues were identified during transfer of specimens between the OR and the microbiology lab.

### Microbiologic investigation

Rhizobium isolates were sub-cultured on several media types including blood agar, chocolate agar, and thioglycolate broth, with growth on all of them, suggesting this was not a primary plate contamination issue. Bacterial sequencing and drug sensitivity testing were not feasible due to limitations of available technology and cost. No growth of Rhizobium occurred upon reprocessing and replating primary tissue samples ground in a new lot of sterile saline nor from the initial body fluid or swab cultures. *Achromonium* fungal species were also isolated from initial tissue specimens in four cases, but not upon reprocessing with sterile saline.

All affected samples were found to have been processed by one laboratory “generalist” technologist, a role that involves clinical specimen preparation across multiple areas of the laboratory and is not an individual who specializes in microbiology specimen processing. Interviews with laboratory personnel and observations of techniques revealed that this technologist used nonsterile saline from a blood bank cube, accessed by a nozzle, to process samples. The technologist had recently modified their technique after inaccurately perceiving that a microbiologist used saline from the blood bank cube to process sterile tissue cultures.

### Patient outcomes

Four of six patients had antibiotic therapy targeted or escalated to address Rhizobium, as the significance of this pathogen had not been determined by the time of their discharge. Two of these four patients (no. 3 and 4 with empyema) required a peripherally inserted central catheter (to complete the recommended course of therapy). No antifungal therapy was administered. All patients tolerated antibiotics well without significant adverse events. No deaths occurred.

## Discussion

The cluster of Rhizobium cases involved in this pseudo-outbreak was interpreted as representing true growth until a probable cause was identified.^5^ Fortunately, no patients were infected with Rhizobium, and no significant harm occurred.

Nonsterile saline has been associated with pseudo-outbreaks of other pathogens including *Burkholderia cepacia* and Legionella and is not unique to Rhizobium.^6,7^ This highlights the need to rapidly pinpoint the outbreak source and identify discrepancies in microbiologic processing and infection control strategies. Laboratory processing errors contributing to pseudo-outbreaks have been described with multi-use reagents, deviating from standard single-use sterile transport mediums to a larger contaminated saline dispensing source, and use of nonsterile pipettes.^8,9^ Specimen processing errors have also occurred among newer or less experienced laboratory members who may be unfamiliar with protocols or systems.^9^ Internal validation of tissue culture processing technique was critical in deciphering the source of culture contamination in our investigation. Unique to some hospitals, particularly with staffing limitations, is the use of laboratory technologist generalists who cross-train with microbiologists to process microbiology specimens. Risk of error under these conditions is likely higher than when specimens are processed by dedicated microbiologists. This event led to revision of the standard operating procedures for tissue processing with clarification to use only sterile saline (rather than an alternate option to use thioglycolate broth for tissue grinding), as well as an in-service performance improvement plan for all laboratory personnel who process tissue cultures. No subsequent cases of Rhizobium have occurred since institution of these actions. Our experience in investigating a cluster of cases with an uncommon pathogen underscores the value of timely, direct communication and collaboration to fully understand and optimize individual techniques and system-level policies and procedures.
